# Supplementary material for: DNA replication in primary hepatocytes without the six-subunit ORC
Source: eLife. 2025 Apr 30;13:RP102915. doi: 10.7554/eLife.102915 (PMC12043314; doi:10.7554/eLife.102915)
Supplement: Figure 1—source data 1. [file elife-102915-fig1-data1.zip › Figure 1-source data 1.pdf]

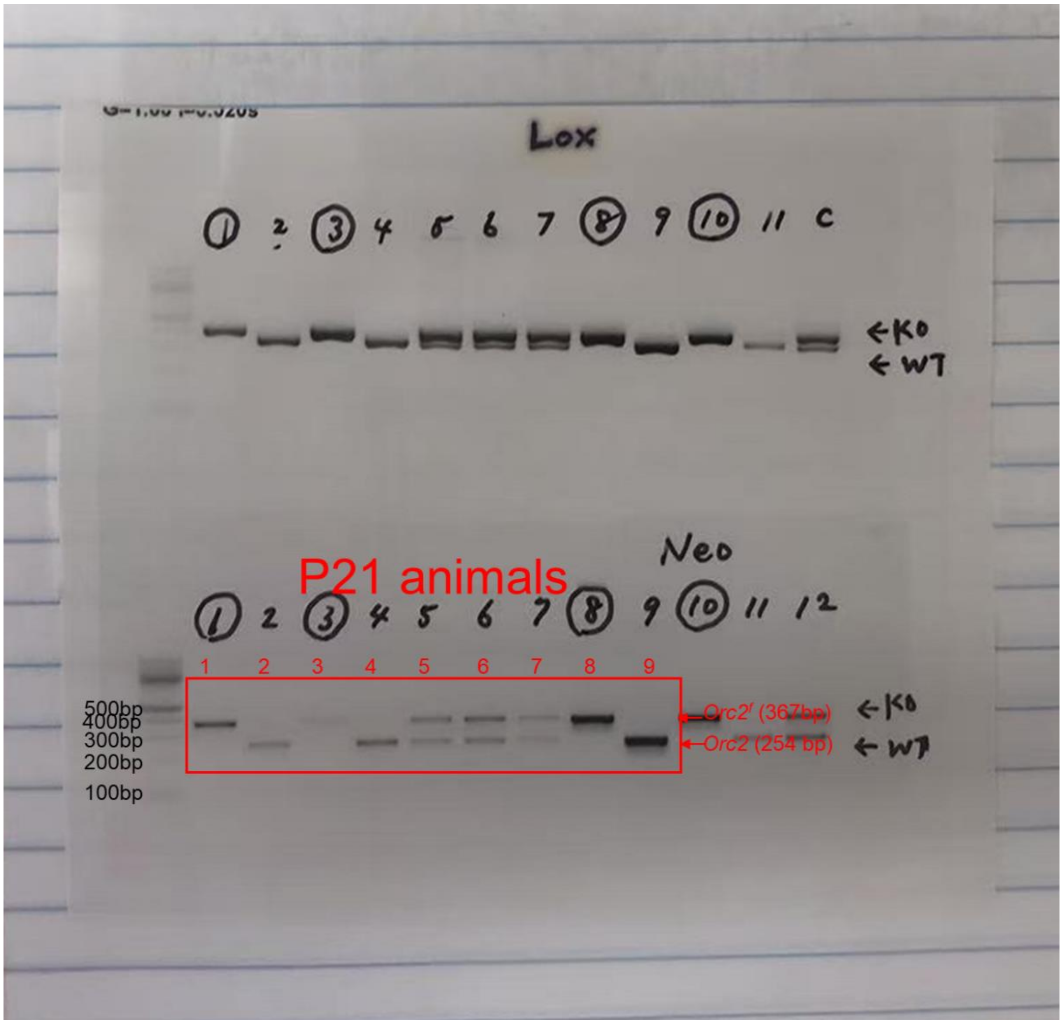

Figure 1, Source Data 1. Original DNA gel picture corresponding to Figure 1, panel B. Bottom gel picture was used for the panel. “Lox” and “Neo” stand for two independent genotyping primers sets. Molecular weight markers are labeled for the appropriate panel. The top band represents mutated allele, the bottom band represents wild-type allele.
