# Supplementary figures and images for: DNA replication in primary hepatocytes without the six-subunit ORC
Source: eLife. 2025 Apr 30;13:RP102915. doi: 10.7554/eLife.102915 (PMC12043314; doi:10.7554/eLife.102915)

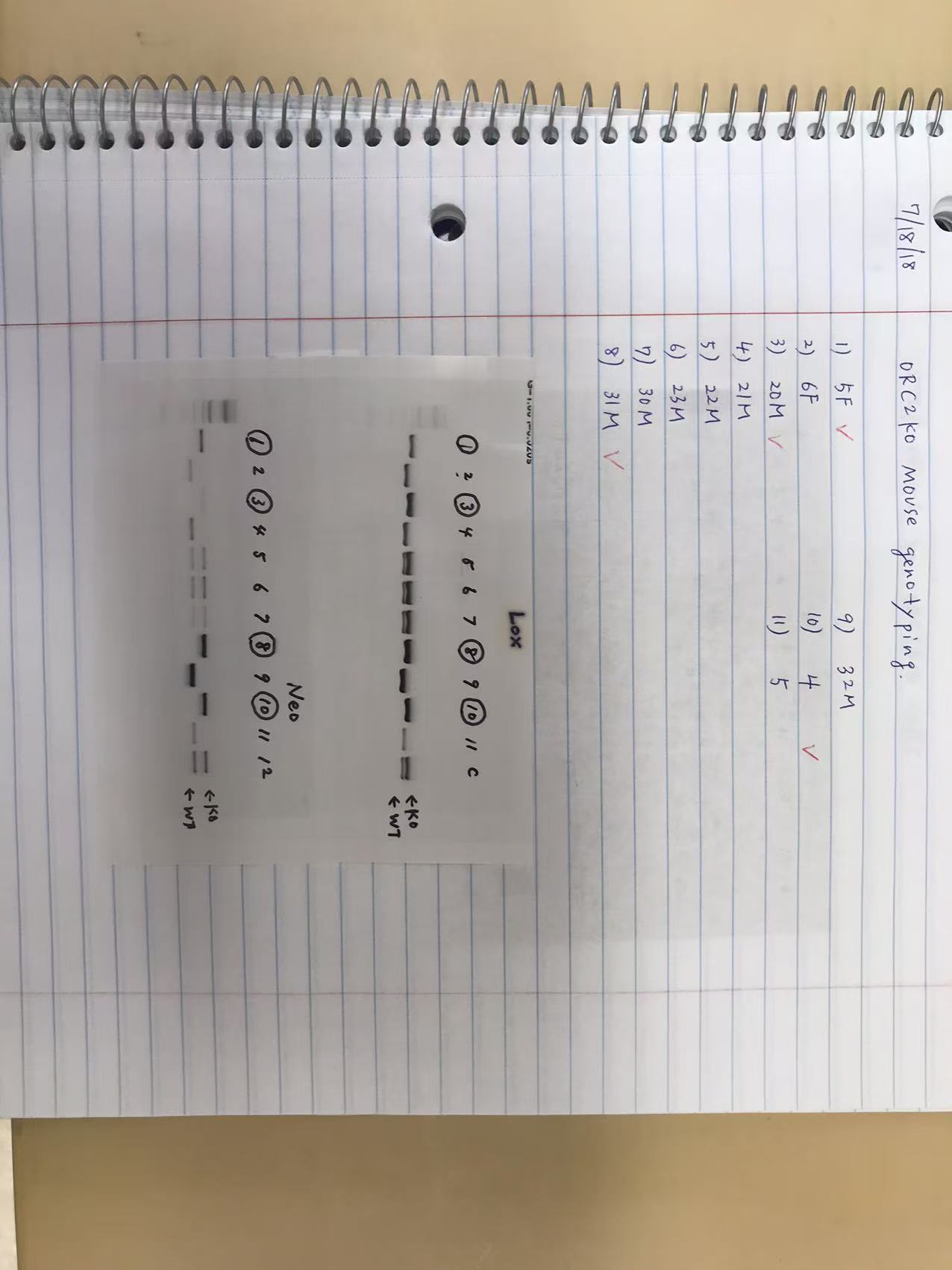

Supplement: Figure 1—source data 2. [file elife-102915-fig1-data2.zip › Figure 1-source data 2.jpg]

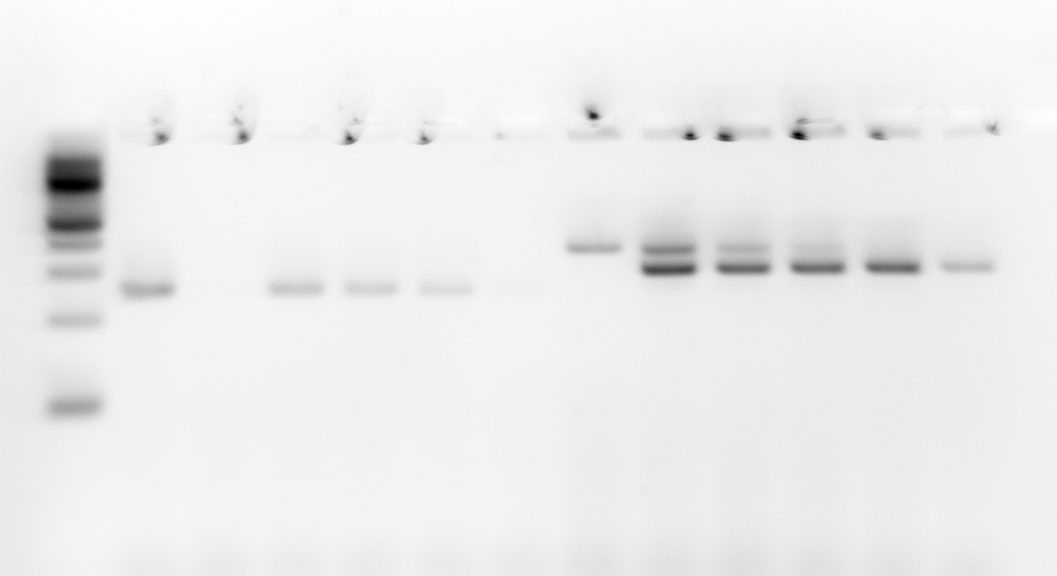

Supplement: Figure 1—source data 4. [file elife-102915-fig1-data4.zip › Figure 1-source data 4.tif]

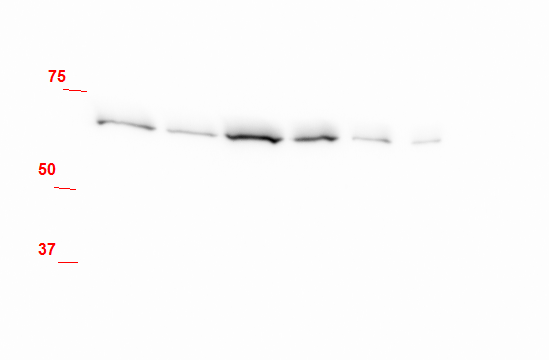

Supplement: Figure 1—source data 6. [file elife-102915-fig1-data6.zip › Figure 1-source data 6.tif]

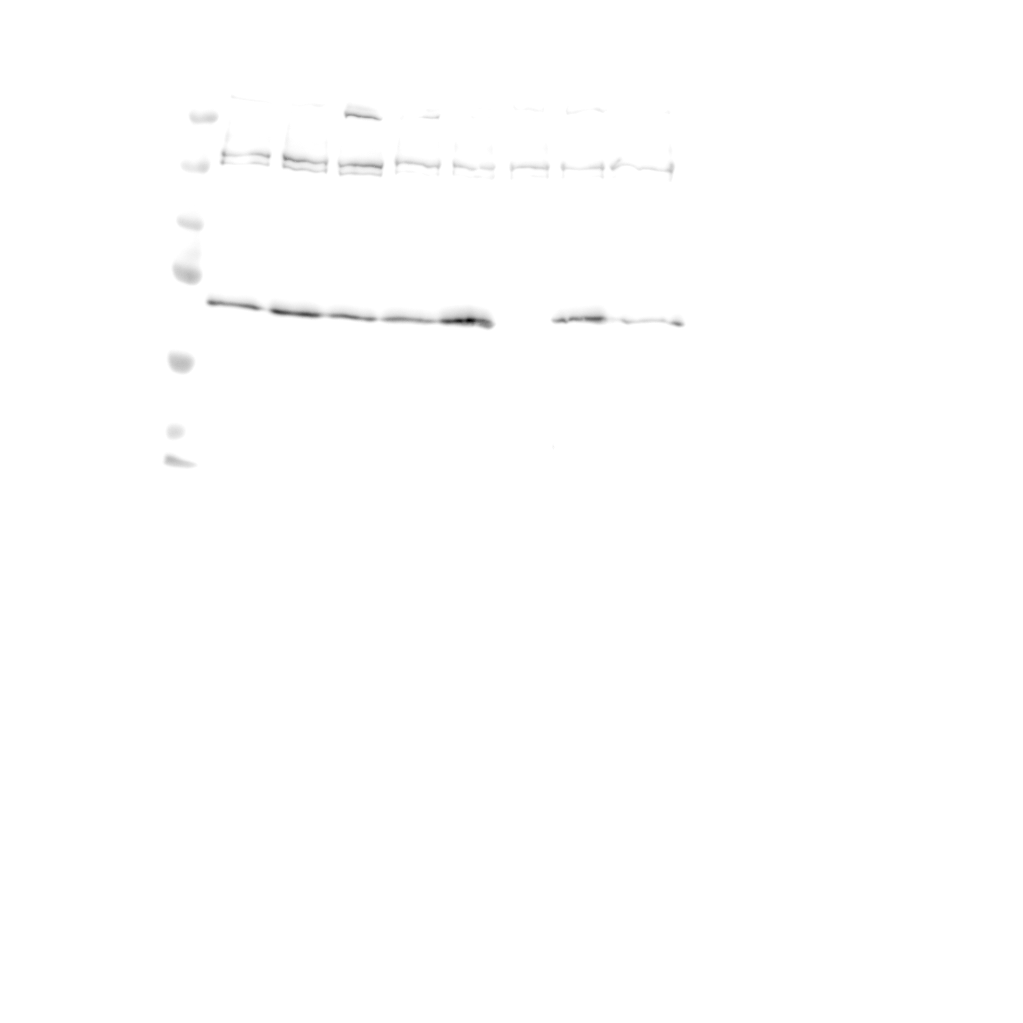

Supplement: Figure 1—source data 8. [file elife-102915-fig1-data8.zip › Figure 1-source data 8.tif]

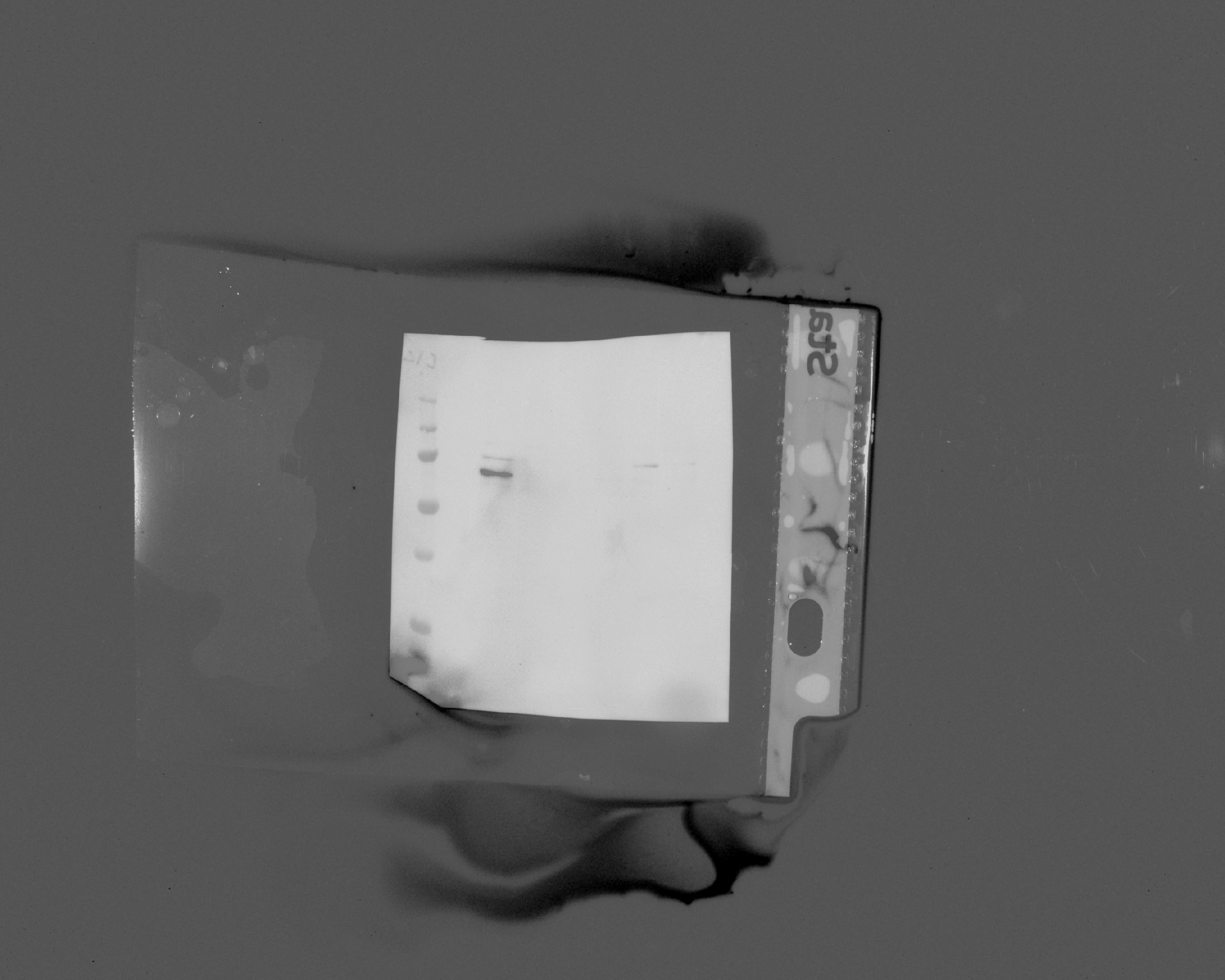

Supplement: Figure 2—source data 2. [file elife-102915-fig2-data2.zip › Figure 2-source data 2.tif]

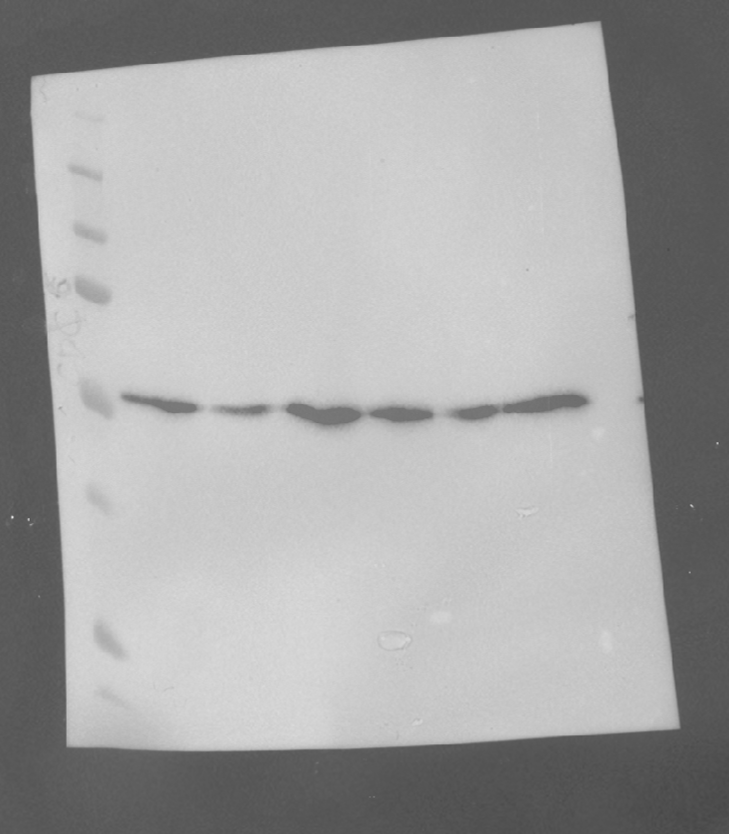

Supplement: Figure 2—source data 4. [file elife-102915-fig2-data4.zip › Figure 2-source data 4.tif]

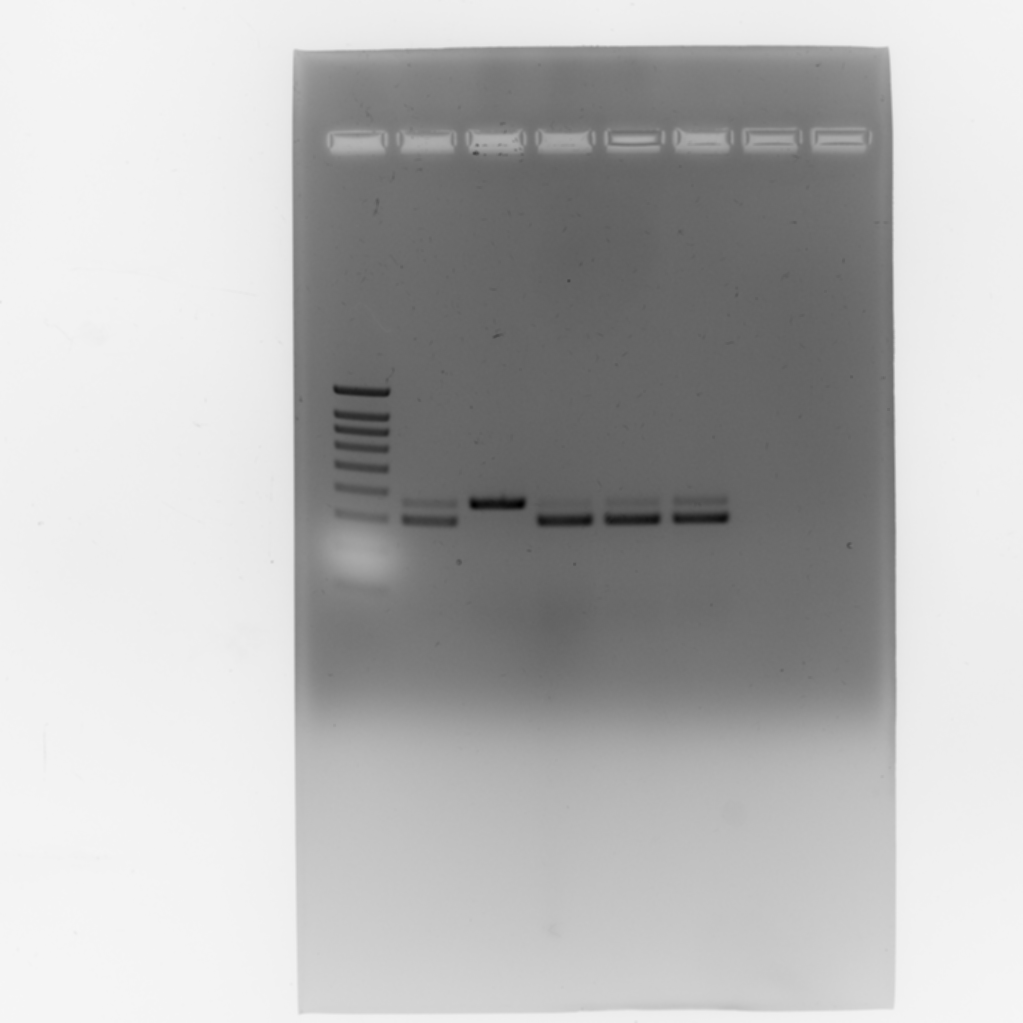

Supplement: Figure 4—source data 2. [file elife-102915-fig4-data2.zip › Figure 4-source data 2.tif]

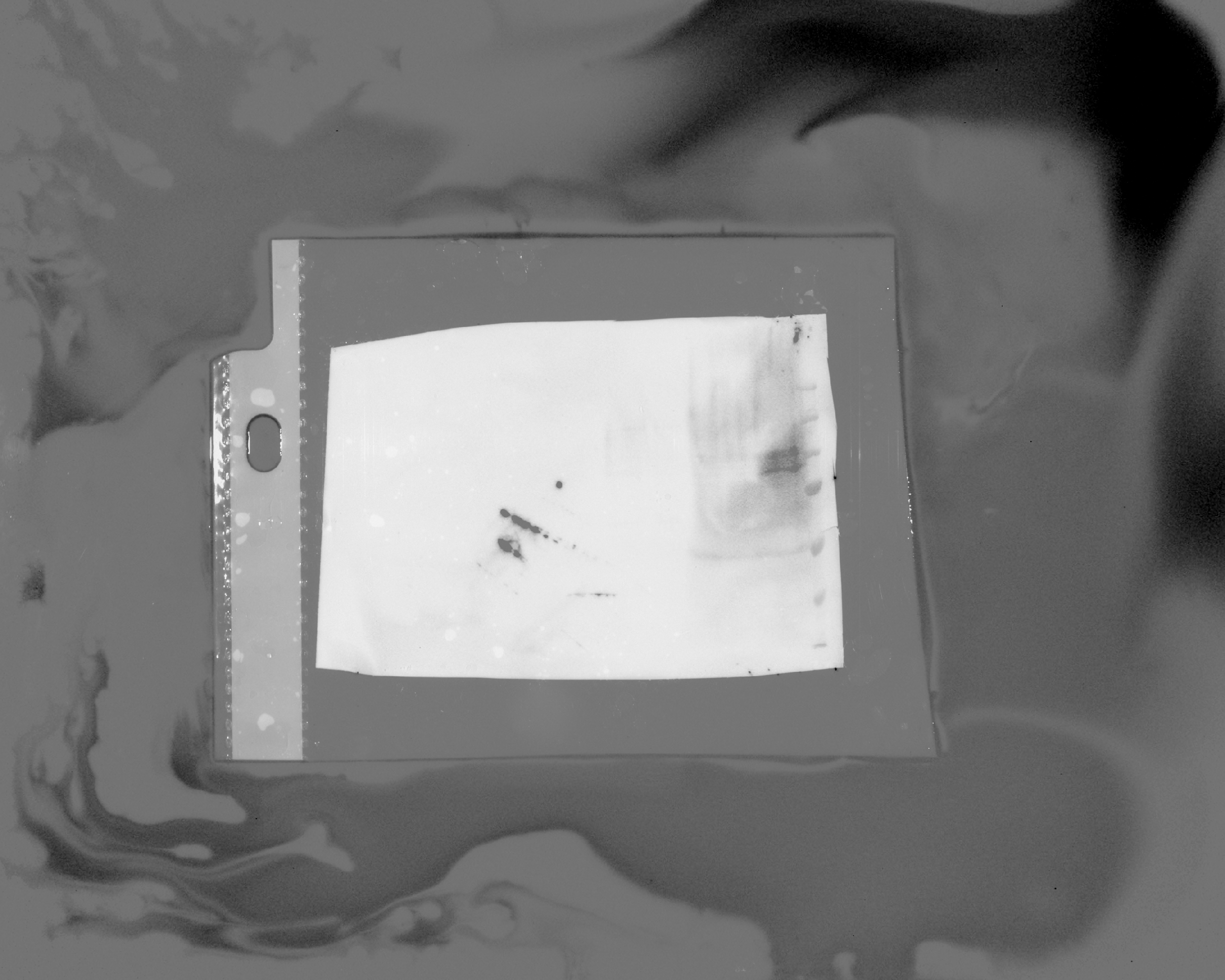

Supplement: Figure 6—source data 2. [file elife-102915-fig6-data2.zip › Figure 6-source data 2.tif]

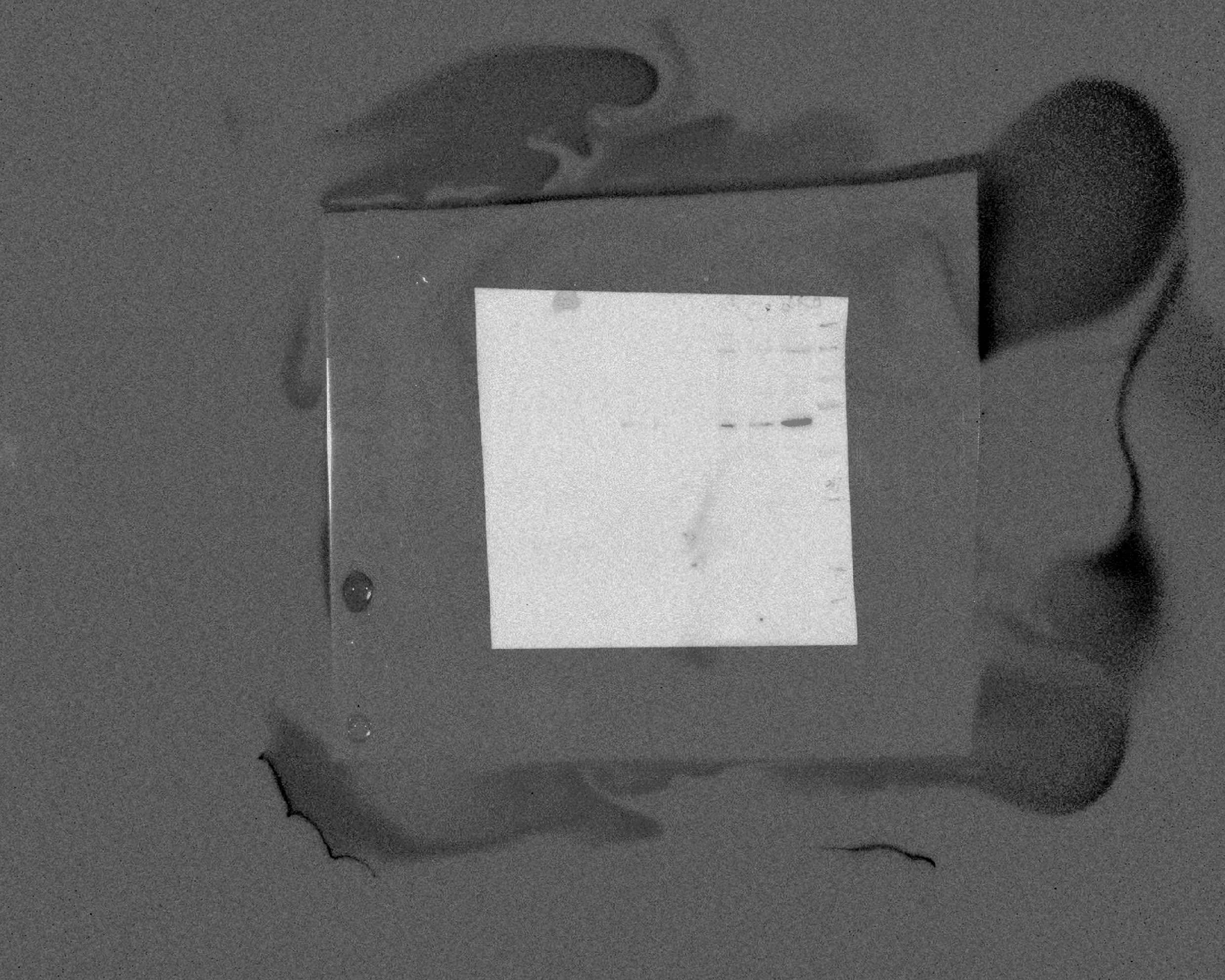

Supplement: Figure 6—source data 4. [file elife-102915-fig6-data4.zip › Figure 6-source data 4.tif]

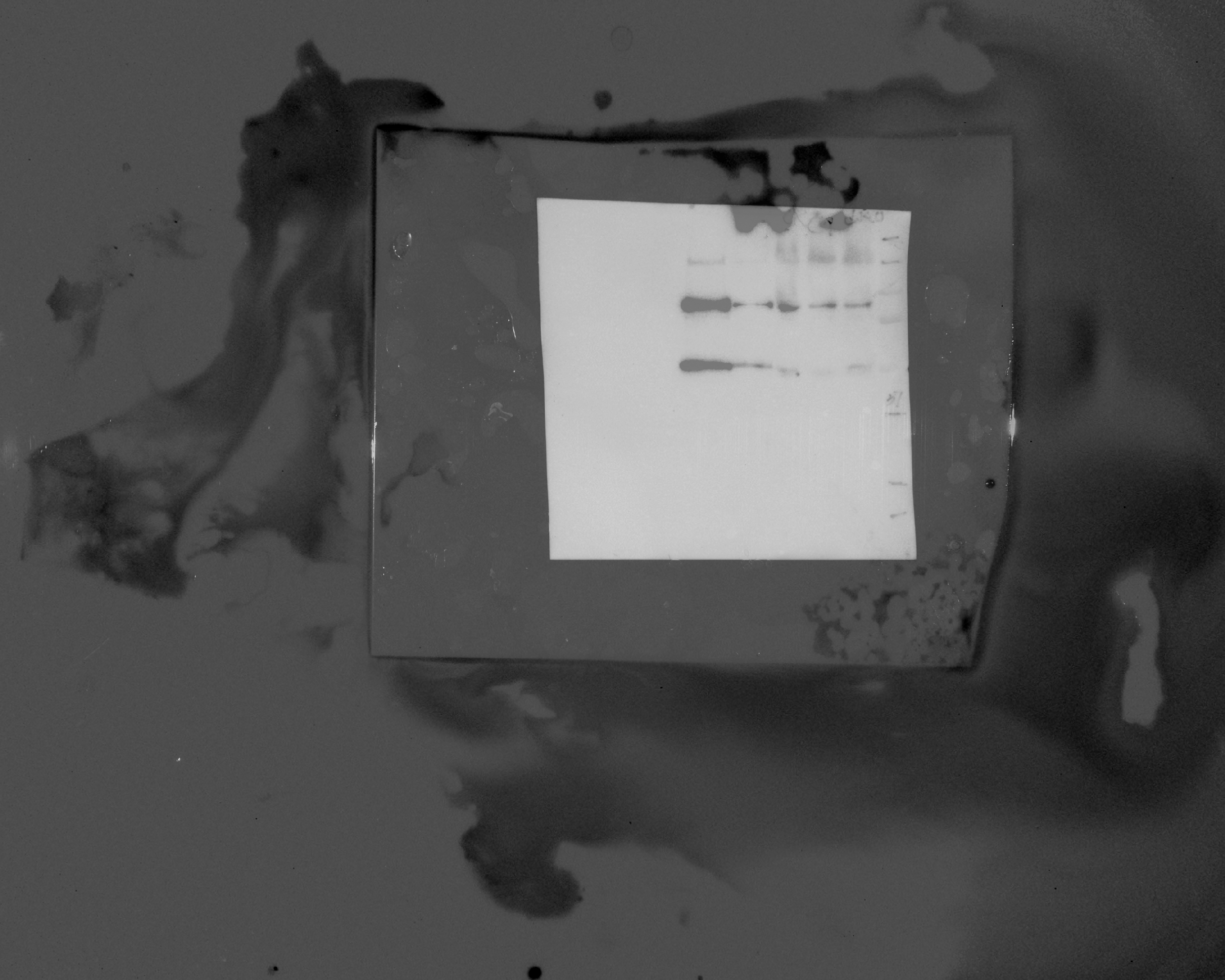

Supplement: Figure 6—source data 6. [file elife-102915-fig6-data6.zip › Figure 6-source data 6.tif]
