# Supplementary material for: DNA replication in primary hepatocytes without the six-subunit ORC
Source: eLife. 2025 Apr 30;13:RP102915. doi: 10.7554/eLife.102915 (PMC12043314; doi:10.7554/eLife.102915)
Supplement: Figure 1—source data 5. [file elife-102915-fig1-data5.zip › Figure 1-source data 5.pdf]

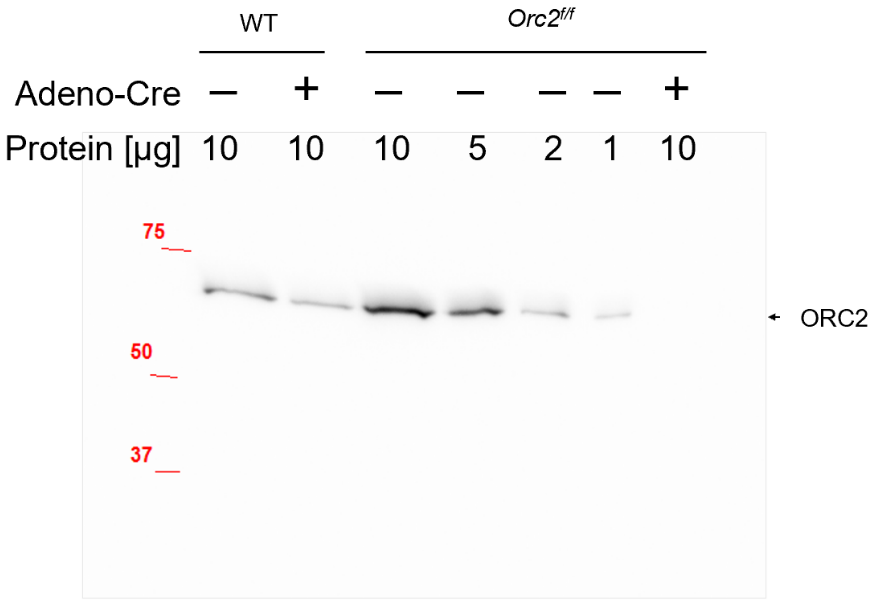

Figure 1, Source Data 3. Original Western blot membrane picture corresponding to Figure 1, panel F. Molecular weight markers are labeled on the left. The band represents ORC2 protein.
