# Supplementary material for: DNA replication in primary hepatocytes without the six-subunit ORC
Source: eLife. 2025 Apr 30;13:RP102915. doi: 10.7554/eLife.102915 (PMC12043314; doi:10.7554/eLife.102915)
Supplement: Figure 2—source data 3. — Molecular weight markers are labeled on the left. The bands next to the arrow represent Tubulin protein. [file elife-102915-fig2-data3.zip › Figure 2-source data 3.pdf]

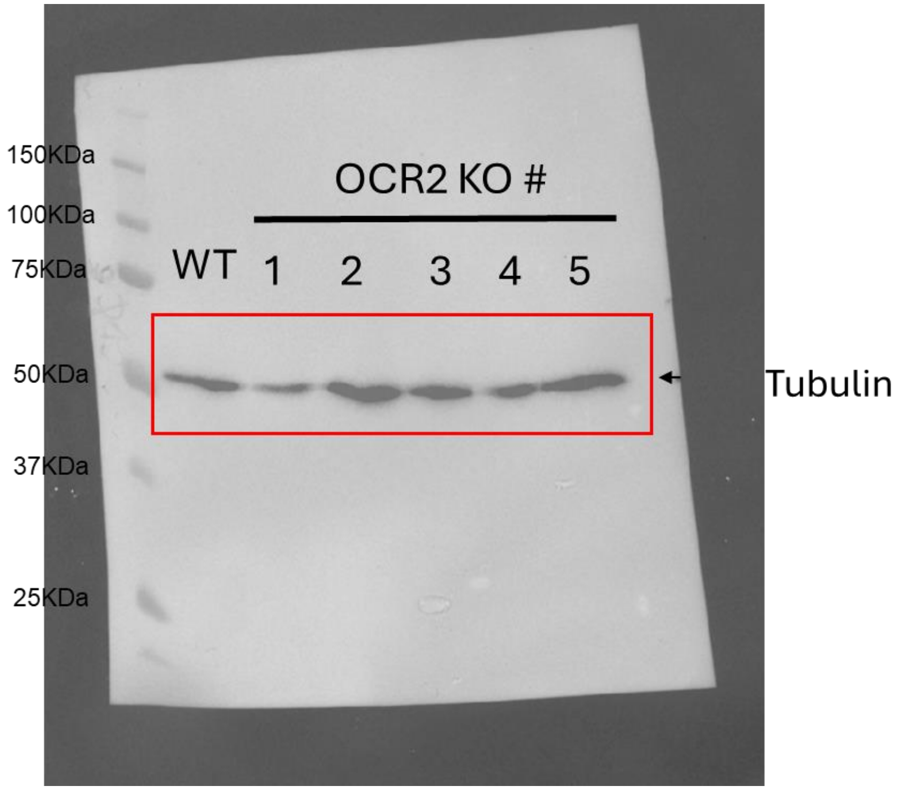

Figure 2, Source Data 2. Original Western blot membrane picture corresponding to Figure 2, panel C. Molecular weight markers are labeled on the left. The bands next to the arrow represent Tubulin protein.
