# Supplementary material for: DNA replication in primary hepatocytes without the six-subunit ORC
Source: eLife. 2025 Apr 30;13:RP102915. doi: 10.7554/eLife.102915 (PMC12043314; doi:10.7554/eLife.102915)
Supplement: Figure 4—source data 1. [file elife-102915-fig4-data1.zip › Figure 4-source data 1.pdf]

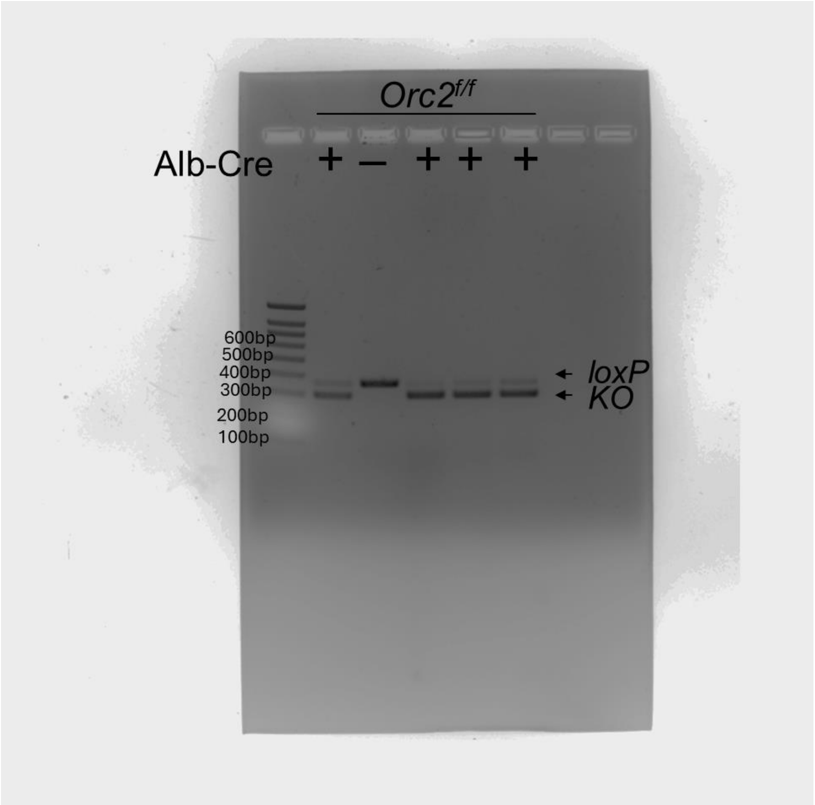

Figure 4, Source Data 1. Original DNA gel picture corresponding to Figure 4, panel B to genotype the hepatocytes. Molecular weight markers are labelled for the appropriate panel. The top band represents Orc2 loxP allele (without Albumin Cre expression), the bottom band represents Orc2 KO allele (with Albumin Cre expression).
