# Supplementary material for: DNA replication in primary hepatocytes without the six-subunit ORC
Source: eLife. 2025 Apr 30;13:RP102915. doi: 10.7554/eLife.102915 (PMC12043314; doi:10.7554/eLife.102915)
Supplement: Figure 6—source data 5. [file elife-102915-fig6-data5.zip › Figure 6-source data 5.pdf]

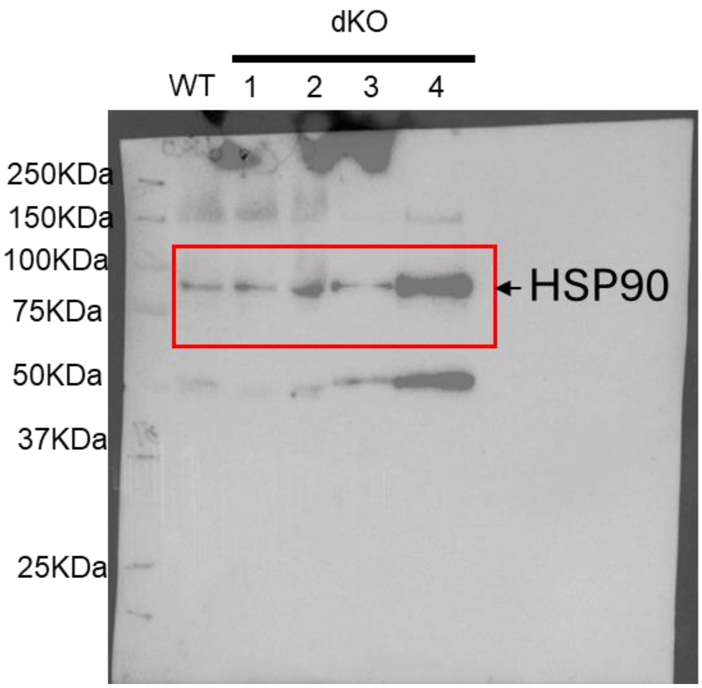

Figure 6, Source Data 3. **Immunoblot of hepatocytes from Orc1$^{f/f}$ Orc2$^{f/f}$ mice (labelled with "WT") and four of Orc1$^{f/f}$ Orc2$^{f/f}$ Alb-cre+/- mice (labelled with (dKO #1-#4)). Protein molecular weight ladder was labelled in the left. The band next to the arrow indicates the HSP90 protein expression level.**
